# The collisional geometry of economical walking predicts human leg and foot segment proportions

**DOI:** 10.1098/rsif.2022.0800

**Published:** 2023-03-22

**Authors:** James R. Usherwood

**Affiliations:** Structure and Motion Lab., The Royal Veterinary College, North Mymms, Hatfield, Herts AL9 7TA UK

**Keywords:** gait, leg, walk, knee, ankle, toe

## Abstract

Human walking appears complicated, with many muscles and joints performing rapidly varying roles over the stride. However, the function of walking is simple: to support body weight as it translates economically. Here, a scenario is proposed for the sequence of joint and muscle actions that achieves this function, with the timing of muscle loading and unloading driven by simple changes in geometry over stance. In the scenario, joints of the legs and feet are sequentially locked, resulting in a vaulting stance phase and three or five rapid ‘mini-vaults’ over a series of ‘virtual legs’ during the step-to-step transition. Collision mechanics indicate that the mechanical work demand is minimized if the changes in the centre-of-mass trajectory over the step-to-step transition are evenly spaced, predicting an even spacing of the virtual legs. The scenario provides a simple account for the work-minimizing mechanisms of joints and muscles in walking, and collision geometry allows leg and foot proportions to be predicted, accounting for the location of the knee halfway down the leg, and the relatively stiff, plantigrade, asymmetric, short-toed human foot.

## 1. Introduction

Human legs and feet are highly derived, presumably adaptively specialized structures. Since the common ancestor with the great apes, leg length has increased in absolute and relative terms, the leg in stance has become relatively straight, feet have become stiffer and toes relatively shorter (e.g. [1]). These changes must surely relate to specialization toward some aspect of terrestrial locomotion; while humans *can* climb, swim and dig, their morphology can hardly be considered specialized for any of these modes. Further, while the abilities of humans at endurance running, and the spring-like action of the human foot arch, has been highlighted [2], the distal limbs of runners ranging from ostrich to horse to modern Paralympic sprinter display feet that are highly elastic, but of relatively low inertia, and much more obviously adapted or designed for running than human feet. The focus here is therefore on interpreting basic aspects of leg and foot function and geometry in the context of economical bipedal walking.

**Figure 1.**
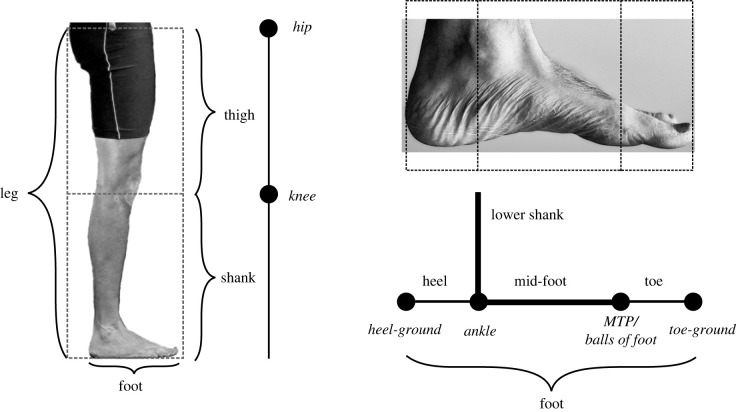
Leg and foot segment and joint (italics) terminology. Dashed boxes indicate exact ½, ½ proportions of the author's leg; or exact ¼, ½, ¼ proportions of the whole of the author's left foot. The author's proportions should not be viewed as statistically representative; however, there is no reason to believe they are other than broadly typical. These exact proportions are predictions of the scenario and are not empirical inputs; they are shown here for reference.

Consideration of animal locomotor mechanics can be pursued at a number of levels, from the highly simplifying point-mass models [3–10] right through to the detailed musculoskeletal analyses (e.g. [11–17]). While the more detailed approaches clearly have merit in some contexts—in particular some ‘how’ questions of muscle contributions when they cannot be measured directly, and ‘what if’ questions demanded when surgical interventions are being modelled—their reliance on many measured inputs make them less well suited to other ‘how’, and the basic ‘why’ questions. Why is the human knee approximately halfway down the leg? Why is the ankle approximately quarter of the way along the foot? And why do we maintain—albeit very much shortened—toes, again of approximately quarter foot length? (figure 1, which also shows the key anatomical terms as used here). Further, how can the apparently complex and rapid sequencing of muscle actions be controlled? Might timing aspects of reflex contributions to muscle activation be driven by simple changes in geometry?

The aim here is to present a scenario for human walking based on work-minimizing principles with physiological constraints, highlighting the role of geometry in loading and unloading various muscles, thereby locking and unlocking a series of joints. It is *not* intended to be a complete model—in particular, it does not predict details of powering. It *is* intended to emphasize—and somewhat artificially separate out—the geometric, passive aspects of economical weight support during walking translation.

## 2. Background and assumptions

### 2.1. Principles of mechanical work and power avoidance—whole limbs and muscles

Mechanical work is the dot product of force and displacement; mechanical power is the dot product of force and velocity. When displacement or velocity is directly in line with the force, the calculation is a simple multiplication. In this case—as applies to a muscle contracting under tension—zero mechanical work or power is supplied by the muscle, either if there is no length change when under load (the muscle is isometric), or when there is no tension during a length change. While the situation becomes complicated when converting to a metabolic power due to the costs of ‘producing’ (equivalently, ‘opposing’) a force, or when the effects of some in-series elastic structure (a tendon) is included, the approximation used here is that isometric muscle produces no work or power and is functionally costless.

In cases where the displacement or velocity are not in line with the force, the calculation is not a simple multiplication: the angle between the two matters. An important limit is when they are perpendicular, as is the case for ideal sliding, rolling, vaulting or pendulum swinging. When perpendicular, the dot product, and work and power demanded, is zero. Mechanisms that allow this state to be approached in high-speed gaits—where forces are predominantly vertical and velocities are approximately horizontal—have been described for mammalian hind- and fore-limbs [18] and are supported by gross anatomy and reported muscle activation sequencing measured with electromyography. The scenario presented here continues with the concept that aspects of limb form and function can be understood from principles of work and power avoidance in terms of both muscle supply and mechanical demand. Can muscles be isometric when loaded, or unloaded when not isometric; and can limbs maintain forces approximately perpendicular to velocities? Unlike the high-speed gaits, fluctuations in height and horizontal velocity during walking are not negligible. However, the principles still apply, obviously during the ‘vaulting’ or ‘inverted pendulum’ phase of stance, but also during the step-to-step transition.

When considering the mechanical power applied to the centre of mass (rate of change of ‘external work’), the relevant forces and velocities relate to the centre of mass (net, including all legs) forces and velocities. When considering the mechanical power of the legs (rate of change of ‘limb work’), each leg force applied to the hip is considered separately, and the relevant velocity is of the hip. No distinction is made here between centre-of-mass ‘external work’ and ‘limb work’ as the hips are co-located and, during periods of double contact, the impulses from each leg are orientated in the same direction (as will be clear from the derived geometries), meaning that the two supporting legs are not putting/taking mechanical work into/from each other. Further, other forms of ‘internal work’ due to motions of masses about the centre of mass are neglected; the legs are assumed to be functionally massless.

### 2.2. The collisional analysis and the implications of finite muscle power

In cases where changes in velocity can be considered as approximating discrete steps, collision mechanics can be convenient and revealing. For a thorough introduction to its application to terrestrial animal locomotion, see [7]. Many of the principles relating to the energetics of collisions are apparent from the principles concerning work of the previous section: low work is demanded for a series of small changes in velocity angle (i.e. the forces are maintained perpendicular to velocity); if velocity is low (as in the hand-hand transition of an idealized, slowly brachiating gibbon, [19]); or if the force is low (as along the arm of a ballistic gibbon, or leg of a running human during the aerial phase). Two key principles come from the collisional analysis: for a given total change in velocity direction, less kinetic energy is lost (and therefore less mechanical work supply is demanded) if it is split into more, smaller, changes in velocity direction than fewer, larger deflections; and for a finite number of heading changes, least work is lost if they are spread evenly, with consistent angular deflections. The analogy of a rimless wheel—a ‘wheel’ with star-like spread of spokes but no outer connections—is sometimes helpful: a rimless wheel can maintain rolling down a shallower (lower power) slope if (i) it has more spokes; and (ii) the spokes are evenly distributed.

An important result from previous collisional analyses of walking [6,7] should be addressed. The mechanical work demand of a stance-to-stance transition is highly dependent on the geometry of the change in velocity from the end of one vaulting stance to the beginning of the next. And it is demonstrated that various strategies involving an additional, powering impulse at the end of stance can advantageously redirect the centre-of-mass velocity just prior to the subsequent, dissipative collision at the beginning of the next. This finding is highly revealing: the details of timing and geometry have a large influence on the mechanical work demands. However, strategies of work-avoiding geometry improvement based on brief periods of high power generation are problematic. Finite work over a brief time requires a high instantaneous power, and muscle power supply is constrained by physiology. So, for a finite positive work demand, brief contraction durations demand very large muscles to be grown, maintained, carried, oscillated and activated—each potentially imposing a real cost for the organism. This issue appears very relevant in biology, accounting for many aspects of deviation from work-minimizing expectations, from the crouched posture of small animals [20], to the gaits of small children [21], to the flapping and bounding of small birds [22]. While the issue of timing may be circumvented to some extent with elastic energy storage and recoil (the Achilles being a prime candidate in humans), it is assumed here that the dissipative power capacity—of not only muscle but also other energy-absorbing tissues—is very much greater than the generating power capacity of muscle, such that a series of plastic (dissipative) collisions achieving low work demand would offer a much lower metabolic and biological cost than a strategy requiring high positive instantaneous power. It should be noted that the proposed scenario of a step-to-step transition based on a series of plastic collisions does not imply net mechanical work dissipation over this period. The scenario does not inform when the work required to overcome the losses should be applied, as long they are not required over a very brief (and therefore high-power) period, or excessively alter centre-of-mass trajectories.

### 2.3. Tension struts, joint limits and sequential vaulting

Examples of tension struts in engineering include bicycle spokes and the cables supporting suspension bridges. These shift the distribution of load-sharing with small changes in geometry, and (being loaded under tension) automatically align the supporting material with the loads—what might be termed ‘anti-buckling’. Motions through stance can cause muscles to act as tension struts, with purely geometric changes suddenly applying load to an isometric muscle, or suddenly unloading the muscle (where, realistically, the muscle is likely to ‘take up the slack’, shortening under low load). In the cases of mammalian fore- and hind-limbs [18], serial loading of two-joint isometric muscles and tendons acting as tension struts form a range of four- and six-bar linkages that result in approximately straight (horizontal) line motion and low mechanical power demand and supply. In the scenario presented here for human walking, the mechanism is a series of vaults, not only over the course of single-legged stance but also the step-to-step transition, created by a series of joint limits. In this idealization, single-joint isometric muscles result in joints becoming suddenly locked when they reach some geometric limit, and freed when the muscle would be loaded in compression. In each vault, the path of the centre-of-mass velocity is perpendicular to a series of ‘virtual legs’ which result from the series of joint limits in one or both legs simultaneously. Following the collisional principles outlined above, the work demand is minimized for the step-to-step transition if the change in direction of each centre-of-mass velocity is even; that is, the ray of virtual legs over this period is evenly spaced.

### 2.4. Two extreme model assumptions: point mass and infinite pitch moment of inertia

The scenario—the sequence of muscle loading and joint locks resulting in low-work step-to-step transition—is presented for two bracketing model assumptions for the pitch moment of inertia of the body. The first presentation assumes a low (zero) pitch moment of inertia. It continuously results in zero net moments about the hip(s), meaning that it would apply to a point mass approximation. The second presentation assumes a very large (infinite) pitch moment of inertia, meaning that moments about the hip have no effect on the pitching motions and energetics of the body. Clearly, neither model is life–like; however, it appears reasonable to assume that predictions consistent for both extremes would offer general insight.

### 2.5. The joints and muscle assumptions

The scenario and models proposed here are planar, with co-located hips. Each leg (figure 1) has pin joints for hip, knee, ankle and metatarsal phalangeal joint (MTP, treated as co-located with the ‘balls of feet’ between mid-foot and toes) and the thigh, shank, heel-to-MTP (heel and mid-foot) and toes are considered rigid elements. The heel-ground and toe-ground contacts are also functional pin joints of the foot. The joints of the foot fall along a line parallel with the sole of the foot. No assumptions are made concerning the relative proportions of each element. For display purposes (only) the leg length used in the figures is approximately realistic, at three times the foot length. Joints may be locked at a limiting angle by tension in isometric single-joint muscles (candidate muscles are named but not asserted, table 1), and these muscles are never longer than their loaded lengths. The heel-ground joint also locks when the sole of the foot contacts the ground. No consideration is made of swinging (protracting) the leg forward.

**Table 1.** The scenario of sequential joint locks resulting in economical walking with a smoothed step-to-step transition. The numbers for the point-mass and infinite pitch moment of inertia models relate to snapshots in figures 2 and 3. Joints are locked either due to muscles coming under isometric tension at their longest length, or by the sole of the foot contacting the ground. Candidate locking tissues are indicated. KCL: knee cruciate ligaments; Sol: soleus; IP: iliopsoas; TF: toe flexor; Gl: gluteus; TA: tibialis anterior; Va: vastus. Briefly rotating joints (*R*) result in a series of vaults that determine the instantaneous centre-of-mass path. Unlocked joints that are not rotating (the remainder) need not require muscle tension (though some degree of stabilization might be expected).

	vault	Joint						model	
		hip	knee	ankle	heel-ground	MTP	toe-ground	point mass (A)	infinite moment of inertia (B)
trailing leg	hip-ankle	*R*		*R*				1	1
	hip-MTP	*R*	KCL	Sol	*R*			2	2
	knee-MTP	IP	*R*	Sol		*R*		3a	3
	knee-toe	IP	*R*	Sol		TF	*R*	4a	4
leading leg	knee-heel	Gl	*R*	TA	*R*			3b	5
	knee-ankle	Gl	*R*	*R*	ground			4b	6
	hip-ankle	*R*	Va	*R*				5	7

## 3. Two models of the scenario

### 3.1. Point mass model (A)

**Figure 2.**
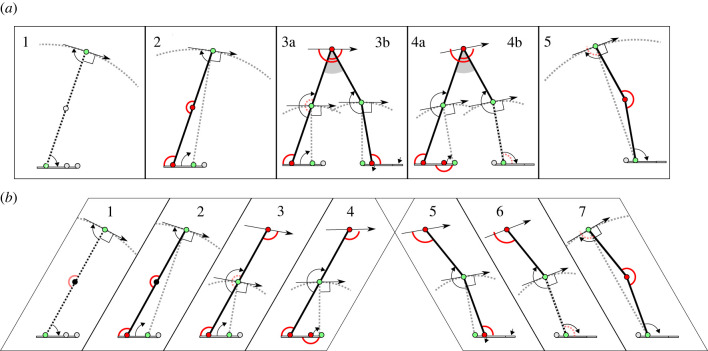
‘Snapshots’ of the step-to-step transition, with a point-mass body (model A) (*a*) and infinite pitch moment of inertia body (model B) (*b*) model extremes. In both cases, the scenario results in a series of ‘mini-vaults’ occurring over a very brief period due to different joint locks (red joint circles), unlocks but not rotating (grey joint circles) and unlocks, rotating (green joint circles) sequenced through geometric changes loading (red arcs) and unloading (dashed, pale red arcs) a series of isometric single-joint muscles. Each muscle is under isometric tension only at its longest length: muscles are engaged and disengaged entirely geometrically. The grey segments (*a*) show the thighs are effectively locked against each other. Each vault and mini-vault is shown with the radius (straight grey dotted lines) and expanded vaulting paths (grey dotted arcs), resulting in a momentary velocity (black straight arrows) perpendicular to the radius. Impulses along each leg are aligned with the radii. Relative motions of the segments (black curved arrows) are considered to be sufficient for the geometric changes in muscle loading, but also very brief, making changes in leg posture over the duration of step-to-step transition otherwise negligible. See the text for description of the stages.

**Figure 3.**
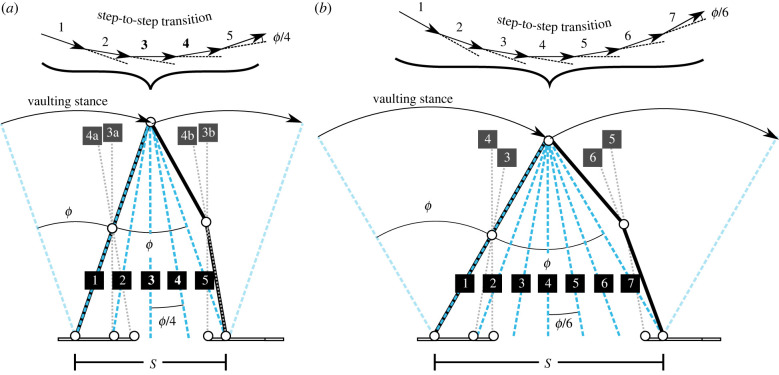
The geometry of economical walking without (*a*, the point mass model (model A)) and with (*b*, the infinite pitch moment of inertia model (model B)) net hip moments. Vaulting stances are separated by a brief step-to-step transition. The velocity and ‘virtual leg’ (blue dashed lines) numbering correspond to snapshots (figure 2, table 1, text). The virtual legs for A**3** and A**4** (bold) touch the ground between the feet; this is achieved with double stance and the 4-bar linkages in 3a, 3b; 4a, 4b (figure 2). The virtual legs for B3–B6 also fall between the feet but do not exploit double stance; they require net hip moments. The kinetic energy dissipation, or negative mechanical work, of a series of collisions over the transition is minimized with even changes in the direction of the velocity vector, and so (with small angle approximations) an even spread of virtual legs. The series of locked joints of the scenario splits a change in angle *ϕ* evenly into four *ϕ*/4 (*a*) or six *ϕ*/6 (*b*) deflections if: one step length *S* is equal to two (*a*) or three (*b*) foot lengths; the knee is halfway down the leg; the heel and toe lengths are equal to *S*/8 (*a*) or *S*/12 (*b*); and the mid-foot segment is *S*/4 (*a*) or *S*/6 (*b*). No account is taken of how energy is put into the cycle, nor the mechanism by which the flexed leg straightens over the vaulting stance. The figure is constructed with the approximate empirical proportions that one leg length equals three feet; but otherwise, the proportions come only from collisional work minimization.

The scenario modelled for a point mass body (figures 2*a* and 3*a*; table 1), precluding any instances of net hip moments, follows through five snapshots over a brief time course of step-to-step transition from one vaulting stance to the next. The geometric collisional development assumes that this sequence happens instantaneously, meaning that the leg segment geometries change minimally, but at the same time are sufficiently spaced such that different muscles are loaded and joints locked. It makes small angle approximations, such that sin⁡ϕ=ϕ and cos⁡ϕ=1. This development supposes that the changes in centre-of-mass velocity follow collisional work-minimizing principles, with four, equal, changes in centre-of-mass (CoM) velocity direction. For a stance angle ϕ (figure 3), leg and foot segment proportions are derived that result in five virtual legs, and four changes in velocity direction from downward (at −ϕ/2) to upward (at +ϕ/2 above horizontal).

#### 3.1.1. Snapshot A1

End of main, straight-legged vaulting stance, with hip rotating about ankle. Centre-of-mass velocity angled downward at −ϕ/2. Moments about the knee are negligible.

#### 3.1.2. Snapshot A2

Ankle locks as muscle (potentially soleus; table 1) suddenly reaches length limit, resulting in straight-legged vault of hip about the MTP, forming a new virtual leg. Moments imposed about the knee are opposed by tissues preventing hyperextension (potentially the knee cruciate ligaments). The centre of mass then momentarily travels perpendicular to this virtual leg during the first ‘mini-vault’. For this change in velocity direction to be +ϕ/4, for a step length *S*, the MTP is located *S*/4 ahead of the ankle (figure 3).

#### 3.1.3. Snapshot A3

This involves a horizontal hip and CoM path due to momentary vaulting about a vertical virtual leg due to the action of both trailing (3a) and leading (3b) legs. The trailing leg locks at the hip as muscle (potentially iliopsoas) suddenly reaches length limit, preventing the thigh from further rotating about the hip, and the ankle continues to be locked, resulting in rotation of the knee about the MTP. For this to be horizontal, contributing to the next +ϕ/4 change in CoM velocity, the knee is directly above the MTP; given the hip is *S*/2 ahead of the ankle, and the MTP *S*/4, this puts the knee halfway down the leg. This generates large moments about the hip; however, these are cancelled by the action of the leading leg (3b), which locks the opposite thigh about the hip with a muscle (presumably a gluteus) at length limit, preventing the thigh from rotating the other way. In the point-mass model, there can be no net moments about the centre of mass, so this is equivalent to locking the two thighs to each other (grey segment, figure 2*a*, snapshots 3 and 4). This results in vaulting of the knee about the heel as the foot sole is initially angled slightly above the ground and the ankle is locked by a muscle at length limit (presumably the tibialis anterior). For this to result in a horizontal second mini-vault, and also meaning that the moments at the hip cancel, the heel-ground ‘joint’ is vertically below the knee.

#### 3.1.4. Snapshot A4

This final mini-vault results in a slight upward CoM trajectory, again through the contributions of both trailing (4a) and leading (4b) legs. Both legs continue to be locked at the hips, and the moments about the hip continue to cancel. In the trailing limb, as the foot lifts, the MTP locks as some muscle (toe flexor) suddenly reaches its length limit. With the ankle continuing to be locked, this results in momentary vaulting of the knee about the toe-ground joint. This results in a change in velocity direction of ϕ/4 if the toe-ground joint is *S*/8 ahead of the knee (and so the MTP): the required velocity vector is +ϕ/4 above the horizontal, so the radius for the vault is ϕ/4 off vertical; with the height of the knee approximately half leg length (recall small angle approximations), and (from above) the knee located above the MTP at a position S/4, the MTP to toe-ground joint (toe length) is *S*/8. At exactly the same time, the foot of leading leg (4b) becomes flat with the ground, thereby unlocking the ankle by suddenly unloading the locking muscle (tibialis anterior). This results in a momentary vault of the lead-leg knee about the ankle. If the shank between knee and ankle is inclined ϕ/4 off vertical, the trajectory is ϕ/4 above horizontal, matching that of the knee of the trailing leg, and—given both hips are locked—resulting in a centre-of-mass velocity change again of ϕ/4 and again without net hip moments. This shank angle, the placement of the knee halfway along the leg (snapshot 3) and the placement of the heel-ground ‘joint’ directly under the knee (result from 3b) requires that the heel-ground joint is placed *S*/8 behind the lead-leg ankle.

Note that, while snapshots 3 and 4 involve simultaneous loading of trailing and leading limbs, in each case a single impulse is applied to the centre of mass because the subsequent direction is determined by the four-bar linkage consisting of joints at trailing knee, leading knee and (snapshot 3) MTP and heel, or (snapshot 4) toe-ground and ankle (green joints in figure 2). This means that the situation is not indeterminate, and the formal unpredictability of simultaneous collisions (classically exemplified by the break in snooker or pool) is not an issue.

#### 3.1.5. Snapshot A5

The beginning of the leading-leg vaulting stance phase, with knee locked by a muscle (presumably a vastus) suddenly reaching a length limit, and the hip lock released as the thigh rotates under the hip, unloading the gluteus. Rotation continues about the ankle which remains unlocked as the foot is flat on the ground and the soleus limit has yet to be reached. This results in a final change in centre-of-mass velocity direction of +ϕ/4, beginning the upward arc at +ϕ/2 if the ankle is *S*/2 ahead of the hip. A geometric—and energetic—inconsistency caused by the small angle approximations should be highlighted: the ankle of the trailing leg is placed *S*/2 behind the hip, and that of the leading leg *S*/2 ahead of the hip, (and the hips are co-located, so are of the same height) but the leading leg is somewhat flexed. *Something additional* is required to restore the flexed stance leg to straight before the end of stance. Similarly, *no account has been taken* of the mechanism of powering (though, for the point-mass case, powering during single stance can only be achieved through leg extension). The scenario is *not* concerned with the mechanisms of energy input (other than it should not require very high instantaneous powers); it is entirely focused on mechanisms for economical weight support during translation.

### 3.2. Infinite pitch moment of inertia model (B)

Geometric principles including small angle approximations are as for the point mass model A development. The infinite pitch moment of inertia model B development allows—and in no way penalizes—instances of net hip moments. This allows the same scenario of sequenced joint locks induced by geometric loading of isometric muscle to be achieved with seven instead of five snapshots, dividing the change in CoM velocity directions over the step-to-step transition into six even changes of ϕ/6 (figures 2*b*, 3*b*).

#### 3.2.1. Snapshot B1

End of straight-legged vaulting stance, with hip rotating about ankle. Centre-of-mass velocity angled downward at −ϕ/2 (=−3ϕ/6).

#### 3.2.2. Snapshot B2

Ankle locks and the hip begins to vault about the MTP. This time, as there are to be six even changes in velocity direction, the CoM velocity becomes −2ϕ/6, and the MTP is located at *S*/6; given the same ratio of foot length to leg length, this strategy results in longer steps. Knee hyperextension is resisted (presumably by the knee cruciate ligaments).

#### 3.2.3. Snapshot B3

The hip locks, and the knee vaults about the MTP; with an infinite pitch moment of inertia, the hip and centre of mass also take the same velocity direction without requiring double stance. The angle between the velocity direction at B2 (−2ϕ/6) and B4 (horizontal) is split evenly with a knee halfway down the leg, resulting in knee and CoM velocity direction of −ϕ/6.

#### 3.2.4. Snapshot B4

The hip continues to be locked, and the MTP locks, resulting in vaulting of the knee about the toe-ground joint. This results in horizontal motion of the knee and (given infinite pitch moment of inertia) CoM if the knee is directly above the toe-ground joint. This is achieved with toe length=*S*/12 (figure 3*b*).

#### 3.2.5. Snapshot B5

Transition to the leading leg, with hip locked by gluteus, ankle locked by tibialis anterior (as in A3b). The knee arcs briefly about the heel-ground joint, resulting in knee and centre-of-mass velocity becoming inclined at +ϕ/6. This determines the positioning of the heel given also the position of the knee demanded by the geometry of snapshots B6 and B7.

#### 3.2.6. Snapshot B6

The sole of the foot of the leading leg contacts the ground, locking the heel-ground joint and unloading the tibialis anterior, thereby unlocking the ankle. The hip continues to be locked by the gluteus, and the knee (and so CoM) velocity direction is inclined at +2ϕ/6 above horizontal if the shank is inclined back from vertical by the same.

#### 3.2.7. Snapshot B7

The knee becomes locked by vastus tension, resulting in the hip vaulting about the ankle and unloading the gluteus. This begins the vaulting stance at a velocity direction +3ϕ/6 (+ϕ/2), albeit with a slightly flexed leg as discussed for snapshot A5. With ankle of leading leg *S*/2 ahead of the hip, and the leading knee *S*/6 behind the leading ankle to result in the shank inclination of B6, the heel-ground joint placement resulting in B5 is *S*/12 behind the leading ankle (figures 2*b*, 3*b*). Again, the vaulting phase begins with a slightly flexed leg; however, in contrast to the point-mass model, positive mechanical work can be supplied during the vaulting phase through both leg extension and moments about the hip.

### 3.3. Summary of geometric results

The point mass model, with no instants of net hip moments, results in fewer step changes in CoM velocity (four) and shorter step lengths (2*S*) than the infinite pitch moment of inertia model (six changes, 3*S* respectively). However, the anatomical proportions predicted to minimize collisional work demands by resulting in even deflections in CoM velocity direction are the same, with the knee halfway down the leg and heel : mid-foot : toe ratio (figure 1) of 1 : 2 : 1.

## 4. Discussion

A distinction needs to be attempted between (i) a mechanically and physiologically sensible interpretation of, and (ii) a mechanistic and predictive understanding of human walking. This is somewhat challenging as there is a long history of measurement of, and great familiarity with, the walking gait, leg and foot; this study is certainly not beginning at absolutely first principles. Very rapid leg protraction would allow very short stances and very small *ϕ*, resulting in very low collisional work demands; presumably these are precluded by some aspect of swing-leg mechanics that is not considered here. Even for steps of finite stance length, there is a range of potentially superior strategies that are not considered here: very many joints, and/or very large, rigid, convexly curved feet could, theoretically, result in walking with lower mechanical work losses (see [7,23]; I also gratefully acknowledge A Ruina & AD Kuo 2022, personal communication at Dynamic Walking, Madison, WI, USA). A full exploration of why many of the theoretically superior possibilities are not observed in nature falls beyond the scope of this paper (though strategies relying on very high instantaneous powers have already been addressed). Candidate issues include the availability of very stiff substrates, evolutionary and developmental constraints, and the advantages of generalism and adaptability. The leg and foot is therefore simply taken as consisting of a limited number of joints: hip, knee, ankle, MTP, heel-ground and toe-ground. With these, the scenario presents a principled framework with clear predictions. But it would be awkward to present these predictions as testable hypotheses, as many of them were familiar before the development of the scenario.

### 4.1. Kinematics

Aspects of easily observed kinematics are clearly consistent with the scenario: all kinematic aspects of the snapshots (figure 2) are immediately familiar in healthy adult human walking, and loss of any joint action often attributable to pathology. The scenario views the snapshots as instantaneous but sequenced. When observing or measuring kinematics, the sequencing may not be apparent; the transitions are not discrete, and there is some overlap between phases. It should be noted that the motions described here have some correspondence with the ‘three rockers of gait’ (e.g. [24]), but are different in detail, mechanism and significance. Similarly, while having some similarities with the ‘six determinants of gait’ [25], the scenario here expressly does *not* suggest advantage to minimizing vertical motions (see [26] for fuller discussion on this topic).

Some explicit qualitative kinematic predictions—for either model presentation of the scenario—that do match human walking include, for the trailing leg: heel lift beginning before heel strike; dorsiflexion about the MTP; and flexion at the knee before toe-off. For the leading leg: a heel strike, with heel behind ankle and with dorsiflexed foot, at the same time as a flexed knee; rapid plantar flexion limited by sole-ground contact; followed by flexion at the knee. Many of these features have alternative or complementary accounts, ranging from, in the trailing leg, initiation of swing (protraction) [27,28], or ankle powering and Achilles tendon elastic energy release [29,30], or both (depending on muscle detail; [27]), to shock or centre-of-mass energy absorption in the leading leg [31]. However, even if each of these aspects are necessary, the scenario provides an account for why they should be achieved in the manners observed—there are many alternative kinematic sequences within which protraction, powering and energy absorption could have been achieved.

The two models of the scenario provide bounding predictions for step length, at 2 or 3 foot-lengths. While step length is variable, certainly as a function of speed, these quantitative predictions do appear sensible: there are 2½ Roman feet to a step, and walking with steps much shorter than 2 feet or longer than 3 does appear awkward and is not commonly observed.

### 4.2. Electromyography

Reported muscle activation timings in walking as measured with electromyography (EMG) are broadly consistent with the proposed scenario, inasmuch as iliopsoas and soleus show a peak in late stance (the trailing leg) and gluteus, tibialis anterior and vastus in early stance (the lead leg) [32]. However, the more detailed sequencing of muscle tensions with late (trailing) or early (lead) stance—in the scenario happening at very nearly the same time but in a specific order—is not immediately apparent in the EMG records.

### 4.3. Leg and foot proportions: a model ‘prediction’

While kinematics and EMG timing are, to some extent, consistent with the scenario presented here, they are also consistent with other interpretations and models ranging from the simplifying collisional [6,7] to the detailed musculoskeletal simulations. But both of these modelling approaches have shortcomings when attempting to interpret structure and function. The extreme reductions have neither much in the way of anthropometric inputs, nor capacity for insights regarding anthropometry (see [33] for criticism of this): they have very few joints, usually treating the leg as a prismatic actuator, or perhaps extending to an ankle and stiff foot; and they take little regard for how muscles are actually arranged. At the other end of the scale, the detailed musculoskeletal simulations take too *much* regard of anthropometry, biasing their findings towards ‘how’ questions over ‘why’. It is true that, with a good cost function of muscle, the timing of activity and contributions to powering of various muscles can (e.g. [34]) be simulated… *given observed anthropometry.* Why do humans walk the way they do? Because it minimizes some aggregated muscle cost function for a given set of bone and muscle geometries. But why those geometries in the first place?

The scenario here effectively falls between the two extremes (though it does fall much closer to the simplifying end of the scale). It does take as given the anthropometric observations that there are two legs, and hip, knee, ankle and MTP joints; but from then on, the kinematics and timing of muscle action come from the principles of mechanical work minimization exploiting the collisional approach. The anatomical geometric results (whether from the short-step point mass, or the long-step infinite pitch moment of inertia models) may therefore be viewed as ‘predictions’ from the scenario: the knee should be halfway down the leg; the heel ¼ foot length; the mid-foot ½ foot length; and the toe ¼ foot length. While no human—nor average of any human population—will meet these predictions exactly, the predictions do appear intriguingly close. The scenario might therefore be viewed as presenting at least an approximate answer to some very fundamental ‘why’ questions concerning not only kinematics (above) but also anatomy. Even if the quantitative results above are viewed as—in detail—inaccurate, the scenario provides a principled, parsimonious account for a range of features of the human leg and foot. The knee should be approximately halfway down the leg; the heel should be shorter than the foot ahead of the ankle (in contrast to some current collisional models, which predict these to be symmetrical; [7,35]), and the toes should be relatively short and capable of some degree of dorsiflexion. While the functional implications of evolutionary reduction, and yet maintenance, of toe length has been considered [36], the scenario appears effective in accounting for a large range of qualitative features of human anatomy through simple energetic and geometric principles; and the quantitative predictions are also encouraging.

### 4.4. A note on motor control

How are the rapidly changing actions of muscles during the step-to-step transition ‘controlled’? At some level there has to be an aspect of ‘top-down’ command: the central control (brain and/or spinal cord) ‘tells’ the legs to keep walking and act appropriately for a given step length [37]. The scenario here, however, emphasizes the other aspect of control: ‘bottom-up’ activation of muscles reacting to loads or length changes [38,39]. Separating the two control strategies may be somewhat arbitrary: there is presumably an interplay between the two. However, the scenario does point towards a means by which the—presumably costly—demands for accurate sensing, interpretation, prediction and top-down signalling, might be ameliorated by discontinuities in kinematics driven by simple geometric changes.

### 4.5. Limitations and opportunities

The reductions necessary for the approach taken here are clearly extreme and mean that many important factors are simply neglected. The mechanical implications of intermediate pitch moment of inertia torso, finite-mass leg segments, visco-elastic material properties and many physiologically important details of neural delays, muscle activation, contraction and cost are completely omitted, and can only be approached with detailed computer simulation. Some—indeed, many—of these features may well account for the deviation between the physics of the scenario described here and anything observed in biology: in reality, there are no discontinuities in any aspects of kinematics or kinetics; joints do not suddenly and completely ‘lock-out’; and the impulses are never observed as infinite forces. However, reductionist linkage and collision mechanics may allow not only insight into several ‘why’ questions—of both kinematic sequencing and anatomical proportions—but also enable ‘what if’ questions to be explored. If the principles and scenario do offer mechanistic insights, then the implications of changes through evolution, pathology and injury might be approached. For instance: what might be expected with a flexible mid-foot? How, then, might the centre-of-mass trajectory be achieved with many, evenly spaced changes in velocity vector?

## 5. Conclusion

The scenario presented here considers reasonable cost functions in walking, explains how this is minimized using collision mechanics, and shows how this is achieved kinematically, through muscles and joints and human-like anatomy and limb proportions. Most notably it may go some way to *explaining* features of the evolved human leg and foot, with knee located halfway down the leg, and the plantigrade foot with short heel behind the ankle, longer mid-foot and stiff, short toes.

## Data Availability

This article has no additional data.

## References

[1] Susman RL. 1983 Evolution of the human foot: evidence from plio-pleistocene hominids. Foot Ankle **3**, 365-376. (10.1177/107110078300300605) 6409715

[2] Bramble DM, Lieberman DE. 2004 Endurance running and the evolution of *Homo*. Nature **432**, 345-352. (10.1038/nature03052) 15549097

[3] Alexander RM. 1976 Mechanics of bipedal locomotion. In Perspectives in experimental biology, Vol. **1** (ed. P Spencer-Davies), pp. 493-504. Oxford, UK: Pergamon Press.

[4] Mochon S, McMahon T. 1980 Ballistic walking. J. Biomech. **13**, 49-57. (10.1016/0021-9290(80)90007-X) 7354094

[5] Alexander RM. 1989 Optimization and gaits in the locomotion of vertebrates. Physiol. Rev. **69**, 1199-1227. (10.1152/physrev.1989.69.4.1199) 2678167

[6] Kuo AD. 2002 Energetics of actively powered locomotion using the simplest walking model. J. Biomed. Eng. **124**, 113-120. (10.1115/1.1427703) 11871597

[7] Ruina A, Bertram JEA, Srinivasan M. 2005 A collisional model of the energetic cost of support work qualitatively explains leg sequencing in walking and galloping, pseudo-elastic leg behavior in running and the walk-to-run transition. J. Theor. Biol. **237**, 170-192. (10.1016/j.jtbi.2005.04.004) 15961114

[8] Geyer H, Seyfarth A, Blickhan R. 2006 Compliant leg behaviour explains basic dynamics of walking and running. Proc. R. Soc. B **273**, 2861-2867. (10.1098/rspb.2006.3637) PMC1664632 17015312

[9] Srinivasan M, Ruina A. 2006 Computer optimization of a minimal biped model discovers walking and running. Nature **439**, 72-75. (10.1038/nature04113) 16155564

[10] Srinivasan M. 2011 Fifteen observations on the structure of energy-minimizing gaits in many simple biped models. J. R. Soc. Interface **8**, 784-798. (10.1098/rsif.2009.0544) PMC3024815 20542957

[11] Anderson FC, Pandy MG. 2001 Dynamic optimization of human walking. J. Biomech. Eng. **123**, 381-390. (10.1115/1.1392310) 11601721

[12] Anderson FC, Pandy MG. 2003 Individual muscle contributions to support in normal walking. Gait Posture **17**, 159-169. (10.1016/S0966-6362(02)00073-5) 12633777

[13] Thelen DG, Anderson FC. 2006 Using computed muscle control to generate forward dynamic simulations of human walking from experimental data. J. Biomech. **39**, 1107-1115. (10.1016/j.jbiomech.2005.02.010) 16023125

[14] Delp SL, Anderson FC, Arnold AS, Loan P, Habib A, John CT, Guendelman E, Thelen DG. 2007 OpenSim: open-source software to create and analyze dynamic simulations of movement. IEEE Trans. Biomed. Eng. **54**, 1940-1950. (10.1109/TBME.2007.901024) 18018689

[15] Liu M, Anderson FC, Schwartz M, Delp S. 2008 Muscle contributions to support and progression over a range of walking speeds. J. Biomech. **43**, 3243-3252. (10.1016/j.jbiomech.2008.07.031) PMC4423744 18822415

[16] Neptune RR, Sasaki K, Kautz SA. 2008 The effect of walking speed on muscle function and mechanical energetics. Gait Posture **28**, 135-143. (10.1016/j.gaitpost.2007.11.004) 18158246 PMC2409271

[17] Umberger BR. 2010 Stance and swing phase costs in human walking. J. R Soc. Interface **7**, 1329-1340. (10.1098/rsif.2010.0084) 20356877 PMC2894890

[18] Usherwood JR. 2022 Legs as linkages: an alternative paradigm for the role of tendons and isometric muscles in facilitating economical gait. J. Exp. Biol. **225**, jeb243254. (10.1242/jeb.243254) 35258605 PMC8987730

[19] Bertram JEA, Ruina A, Cannon EE, Chang YH, Coleman MJ. 1999 A point-mass model of gibbon locomotion. J. Exp. Biol. **202**, 2609-2617. (10.1242/jeb.202.19.2609) 10482720

[20] Usherwood JR. 2013 Constraints on muscle performance provide a novel explanation for the scaling of posture in terrestrial animals. Biol. Lett. **9**, 20130414. (10.1098/rsbl.2013.0414) 23825086 PMC3730652

[21] Usherwood JR, Hubel TY, Smith BJH, Davies ZTS, Sobota G. 2018 The scaling or ontogeny of human gait kinetics and walk-run transition: the implications of work vs. peak power minimization. J. Biomech. **81**, 12-21. (10.1016/j.jbiomech.2018.09.004) 30316545 PMC6224478

[22] Usherwood JR. 2016 Physiological, aerodynamic and geometric constraints of flapping account for bird gaits, and bounding and flap-gliding flight strategies. J. Theor. Biol. **408**, 42-52. (10.1016/j.jtbi.2016.07.003) 27418386 PMC5042028

[23] Adamczyk PG, Collins SH, Kuo AD. 2006 The advantages of a rolling foot in human walking. J. Exp. Biol. **209**, 3953-3963. (10.1242/jeb.02455) 17023589

[24] Mayich DJ, Novak A, Vena D, Daniels TR, Brodsky JW. 2014 Gait analysis in orthopedic foot and ankle surgery – topical review, Part 1: principles and uses of gait analysis. Foot Ankle Int. **35**, 80-90. (10.1177/1071100713508394) 24220612

[25] Saunders J, Inman V, Eberhart H. 1953 The major determinants in normal and pathological gait. Am. J. Bone Joint Surgery **35**, 543-558. (10.2106/00004623-195335030-00003) 13069544

[26] Kuo AD. 2007 The six determinants of gait and the inverted pendulum analogy: a dynamic walking perspective. Hum. Mov. Sci. **26**, 617-656. (10.1016/j.humov.2007.04.003) 17617481

[27] Neptune RR, Kautz SA, Zajac FE. 2001 Contributions of the ankle plantar flexors to support, forward progression and swing initiation during walking. J. Biomechanics **34**, 1387-1398. (10.1016/S0021-9290(01)00105-1) 11672713

[28] Lipfert SW, Günther M, Renjewski D, Seyfarth A. 2014 Impulsive ankle push-off powers leg swing in human walking. J. Exp. Biol. **217**, 1218-1228. (10.1242/jeb.107391) 24363410

[29] Lichtwark GA, Wilson AM. 2007 Is Achilles tendon compliance optimized for maximum muscle efficiency during locomotion? J. Biomech. **40**, 1768-1775. (10.1016/j.jbiomech.2006.07.025) 17101140

[30] Zelik KE, Huang T-W, Adamczyk PG, Kuo AD. 2014 The role of series ankle elasticity in bipedal walking. J. Theor. Biol. **346**, 75-85. (10.1016/j.jtbi.2013.12.014) 24365635 PMC3947753

[31] Usherwood JR, Channon AJ, Myatt JP, Rankin JW, Hubel TY. 2012 The human foot and heel-sole-toe walking strategy: a mechanism enabling an inverted pendular gait with low isometric muscle force? J. R. Soc. Interface **9**, 2396-2402. (10.1098/rsif.2012.0179) 22572024 PMC3427509

[32] Cappellini G, Ivanenko YP, Poppele RE, Lacquaniti F. 2006 Motor patterns in human walking and running. J. Neurophysiol. **95**, 3426-3437. (10.1152/jn.00081.2006) 16554517

[33] Zajac FE, Neptune RR, Kautz SA. 2003 Biomechanics and muscle coordination of human walking. Part II: lessons from dynamical simulations and clinical implications. Gait Posture **17**, 1-17. (10.1016/s0966-6362(02)00069-3) 12535721

[34] Falisse A, Serrancolí G, Dembia CL, Gillis J, Jonkers I, De Groote F. 2019 Rapid predictive simulations with complex musculoskeletal models suggest that diverse healthy and pathological human gaits can emerge from similar control strategies. J. R. Soc. Interface **16**, 20190402. (10.1098/rsif.2019.0402) 31431186 PMC6731507

[35] Croft JL, Bertram JEA. 2020 Form in the context of function: fundamentals of an energy effective striding walk, the role of the plantigrade foot and its expected size. Am. J. Biol. Anthropol. **173**, 760-767. (10.1002/ajpa.24122) 32932555

[36] Rolian C, Lieberman DE, Hamill J, Scott JW, Werbel W. 2009 Walking, running and the evolution of short toes in humans. J. Exp. Biol. **212**, 713-721. (10.1242/jeb.019885) 19218523

[37] Dzeladini F, Kieboob J, Ijspeert A. 2014 The contribution of a central pattern generator in a reflex-based neuromuscular model. Front. Hum. Neurosci. **8**, 371. (10.3389/fnhum.2014.00371) 25018712 PMC4071613

[38] Sinkjaer T, Andersen JB, Ladouceur M, Christensen LOD, Nielsen JB. 2000 Major role for sensory feedback in soleus EMG activity in the stance phase of walking in man. J. Physiol. **523**, 817-827. (10.1111/j.1469-7793.2000.00817.x) 10718758 PMC2269822

[39] Geyer H, Herr H. 2010 A muscle-reflex model that encodes principles of legged mechanics produces human walking dynamics and muscle activities. IEEE Trans. Neural Syst. Rehabil. Eng. **18**, 263-273. (10.1109/TNSRE.2010.2047592) 20378480

